# Use of Complementary and Alternative Medicine and Its Relationship with Health-Related Quality of Life (HRQOL) in Patients with Type 2 Diabetes Mellitus

**DOI:** 10.3390/epidemiologia4010005

**Published:** 2023-01-20

**Authors:** Yoshiro Mori, Hiroyuki Daikuhara, Tomie Oshima, Hiromi Suzuki, Setsuo Okada, Nobuyuki Miyatake

**Affiliations:** 1 Department of Hygiene, Faculty of Medicine, Kagawa University, Miki 761-0793, Japan; 2 Sakaide City Hospital, Sakaide 762-8550, Japan

**Keywords:** type 2 diabetes mellitus, complementary and alternative medicine (CAM), health-related quality of life (HRQOL)

## Abstract

The present study investigated the use of complementary and alternative medicine (CAM) and its relationship with health-related quality of life (HRQOL) in patients with type 2 diabetes mellitus. A total of 421 patients with type 2 diabetes mellitus who completely met the inclusion criteria, aged 67.3 ± 12.8 years, among 622 outpatients were recruited in this cross-sectional study. We examined the use of CAM, such as supplements, Kampo, acupuncture, and yoga. HRQOL was assessed by EuroQOL. A total of 161 patients (38.2%) with type 2 diabetes mellitus used some type of CAM. The use of supplements and/or health foods was the highest among CAM users (112 subjects, 26.6%). HRQOL was significantly lower in patients who used some CAM (0.829 ± 0.221) than in those without any CAM use (0.881 ± 0.189), even after adjustments for confounding factors [F(1, 414) = 2.530, *p* = 0.014]. Proper information on CAM is needed for patients with type 2 diabetes mellitus.

## 1. Introduction

The Ministry of Health, Labour and Welfare, Japan, estimated that the number of patients with and/or suspected of having diabetes mellitus is approximately twenty million [1]. The goal of treatment is to live a life of similar quality to that of a healthy subject, and maintain quality of life (QOL) [2]. Therefore, appropriate treatment strategies for diabetes mellitus are urgently required. Lifestyle modifications, such as diet and exercise, and drug therapy, including insulin, are the main therapeutic approaches for patients with type 2 diabetes mellitus.

Complementary and alternative medicine (CAM) is a generic term for non-mainstream medicine that is used in conjunction with (complementary) or instead of (alternative) globalized and standardized modern medicine [3]. CAM includes Kampo, acupuncture, acupressure, ayurveda, aromatherapy, and spa therapy. In some meta-analysis reviews, many CAM lack experimental evidence to support their effects, while the benefits of some Kampo formulations and acupuncture were only verified by a certain degree of evidence [4,5,6,7,8,9,10]. Previous studies reported that approximately 35% of the population in the USA and 50–80% in Japan used CAM [11,12,13,14,15,16]. However, limited information is currently available on the use of CAM by patients with type 2 diabetes mellitus in Japan [17]. Moreover, the relationship between the use of CAM and health-related QOL (HRQOL) has not yet been examined. Therefore, the effects of CAM on the prevention and attenuation of type 2 diabetes mellitus in Japan warrants further study.

The present exploratory study investigated the use of CAM and its relationship with HRQOL in Japanese patients with type 2 diabetes mellitus.

## 2. Materials and Methods

### 2.1. Subjects

A total of 421 patients with type 2 diabetes mellitus were enrolled in this cross-sectional exploratory study who completely met the following criteria: (1) an outpatient at Sakaide City Hospital, Sakaide, Japan between 12 July 2021 and 17 September 2021, (2) completed a self-administered questionnaire on CAM and HRQOL, (3) received conventional therapy and blood examinations, and (4) provided written informed consent.

Ethical approval was obtained from the Ethical Committee at Sakaide City Hospital, Sakaide, Japan (Ethical number: 2021-003, Date: 31 May 2021).

### 2.2. CAM

The use of CAM was evaluated by a self-reported questionnaire according to the Ministry of Health, Labour and Welfare, Japan [18]. CAM in the questionnaire included the following: supplements and/or health food, massage, osteopathy, spa therapy, aromatherapy, Kampo, acupuncture and/or moxibustion, yoga, bone grafting, chiropractic therapy, magnetic therapy, forest therapy, music therapy, thermotherapy, qigong, fasting therapy, ayurveda, and homeopathy. Subjects selected one of the following 4 answers: (1) I have used and still use it, (2) I have used, but I do not use it now, (3) I have never used it, and (4) I do not remember and/or do not know.

### 2.3. HRQOL

HRQOL was evaluated using the Japanese version of EuroQOL (EQ-5D) [19]. “EQ-5D consists of five subscales with three levels collectively describing 3^5^ (i.e., 243) possible health combinations. The five subscales are (1) mobility, (2) self-care, (3) usual activities, (4) pain/discomfort, and (5) anxiety/depression”. The QOL score reveals a one-dimensional number ranging between −0.594 (0 means death, less than 0 means worse than death) and 1 (full health). Cronbach’s alpha was reported to be 0.75 (preoperative) and 0.88 (postoperative) in patients undergoing primary knee arthroplasty [20], and 0.87 in patients with type 2 diabetes mellitus [21].

### 2.4. Clinical Parameters

The following clinical data were collected from medical records: age (years), height (cm), body weight (kg), duration of diabetes mellitus (days), albumin (g/mL), triglycerides (mg/dL), LDL-cholesterol (mg/dL), HDL-cholesterol (mg/dL), HbA1c (%), blood glucose (mg/dL), serum creatinine (mg/dL), the estimated glomerular filtration rate (eGFR) (mL/min/1.73m^2^), insulin usage, and the number of oral hypoglycemic agents. Body mass index (kg/m^2^) was calculated by body weight (kg)/[height (m)]^2^. Blood examinations were performed using conventional laboratory methods. eGFR was calculated by the following equation: eGFR (mL/min/1.73m^2^) = 194 × Cr ^−1.094^×Age^−0.287^ (if women: ×0.739) [22].

### 2.5. Statistical Analysis

Continuous data were expressed as the mean ± standard deviation (SD). Categorical data were expressed as a number and frequency (%). We evaluated the normality by using Shapiro–Wilk test and used the unpaired *t*-test (normal distribution), and Mann–Whitney *U* test and χ^2^ test (non-normal distribution) to compare between two groups with and without the use of CAM. In addition, an analysis of covariance (ANCOVA) was used to adjust for confounding factors, where *p* < 0.05 was significant. We did not calculate the sample size of this study because this study was an exploratory study. All statistical analyses were performed by JMP15 Pro 15.1.0 (SAS, Cary, NC, USA).

## 3. Results

Table 1 shows the clinical profiles of patients with type 2 diabetes mellitus enrolled in the present study. Mean age was 67.3 ± 12.8 years and HbA1c was 7.5 ± 1.5%. HRQOL was 0.860 ± 0.200.

We examined the use of CAM by patients with type 2 diabetes mellitus (Table 2). Some patients with type 2 diabetes mellitus use CAM. The use of supplements and/or health foods (112 patients, 26.6%) was the highest among CAM users. A total of 161 patients (38.2%) indicated the use of some type of CAM in the survey.

We then compared HRQOL between patients, with and without the use of CAM (Table 3). The group was divided into two groups as follows: CAM (+) (I have used and still use) and CAM (−) (others). HRQOL was significantly lower in patients using than in those not using spa therapy, Kampo, chiropractic therapy, magnetic therapy, and thermotherapy. We were unable to evaluate HRQOL between patients with and without forest therapy and ayurveda because only one patient used these CAM.

We examined and compared clinical profiles between patients with and without the use of CAM (Table 4). A significant difference was observed in sex, but not in clinical parameters between the two groups.

**Figure 1:**
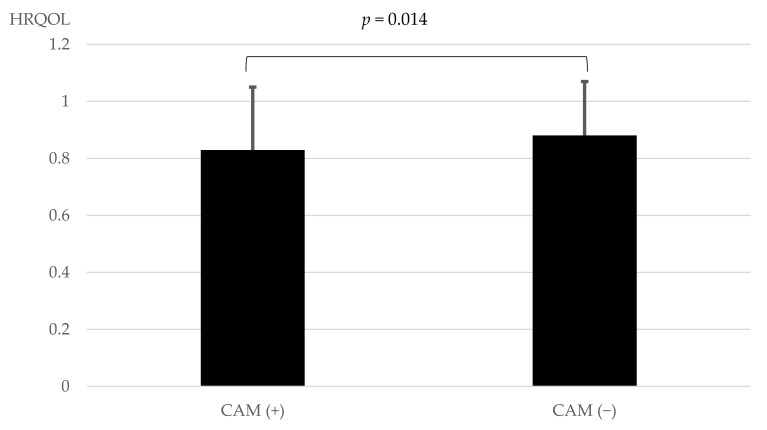
Comparison of HRQOL between patients with and without the use of CAM. Statistical analyses were performed using an analysis of covariance (ANCOVA) with adjustments for sex, age, BMI, the duration of diabetes, and HbA1c. HRQOL: health-related quality of life. CAM: complementary and alternative medicine.

A comparison of HRQOL between patients with and without the use of CAM is shown in Figure 1. HRQOL was significantly lower in patients (0.829 ± 0.221) with than in those without the use of CAM (0.881 ± 0.189) after adjustments for sex, age, BMI, the duration of diabetes, and HbA1c [F(1, 414) = 2.530, *p* = 0.014] (Figure 1).

## 4. Discussion

The present results revealed that approximately 38% of patients with type 2 diabetes mellitus used CAM, and HRQOL was significantly lower in patients with the use of some CAM than in those without the use of any CAM, after adjustments for confounding factors.

Eisenberg et al. previously reported that 39.8% (1990) and 38.5% (1997) of subjects who participated in a telephone survey in the USA used CAM [11]. Clarke et al. showed that 32.3% (2002), 35.5% (2007), and 33.2% (2012) subjects who completed a web-based survey used some type of CAM in the past 12 months [12]. In Japan, 60.9% of adults aged 20–69 years who responded to a postal survey used CAM [13]. Other studies revealed that 77.4% [14], 76% [15], and 57% [16] of their subjects used CAM. Radwan et al. found that 39.2% of patients with type 2 diabetes mellitus in UAE who completed a self-reported questionnaire used CAM [23]. Yıldırım et al. also showed that 36.7% of Turkish patients used CAM, and the rate of CAM users was significantly higher in women than in men [24]. In Japan, Mukai et al. reported that 39.1% of patients who completed a self-reported questionnaire used CAM [17].

In the present study, approximately 38.2% of patients with diabetes mellitus used some type of CAM, and the use of supplements and/or health foods (26.6%) was the highest among CAM users, followed by massage, osteopathy, and Kampo. These results are consistent with previous findings on patients with type 2 diabetes mellitus [10,23,24]. The present study provides useful information on the use of CAM by Japanese patients with type 2 diabetes mellitus.

Previous studies reported a relationship between the use of CAM and HRQOL [25,26,27,28]. Huo et al. evaluated HRQOL in 17,923 patients with bronchial asthma using the Behavioral Risk Factor Surveillance System, and showed that HRQOL was significantly lower in patients with than in those without the use of CAM [25]. Opheim et al. also demonstrated that HRQOL was significantly lower in inflammatory bowel disease (IBD) patients with than in those without the use of CAM [26]. These findings indicate that the use of some CAM is associated with lower HRQOL. Consistent with previous findings, HRQOL was significantly lower in patients with the use of some CAM than in those without any CAM in the present study. Patients with lower HRQOL may require CAM in addition to conventional therapy. Although HRQOL was improved by CAM in adult cancer patients [27], a longitudinal analysis revealed that CAM users did not moderate HRQOL [28]. Due to the limited amount of evidence currently available to support the benefits of CAM [4,5,6,7,8,9,10], further studies are warranted on the use of CAM by patients with type 2 diabetes mellitus in order to provide appropriate advice that will ultimately improve QOL in clinical practice.

There are a number of limitations that need to be addressed. This was a cross-sectional, not longitudinal analysis. Therefore, the direction of causality was not clear. It could be speculated that patients using CAM have lower QOL, not because of CAM use, but because of the presence of comorbidities which led to CAM use. Furthermore, enrolled patients with type 2 diabetes mellitus were considered to be more health conscious than other patients with type 2 diabetes mellitus. Moreover, the present study was performed at one hospital in Sakaide, Japan. Therefore, the results obtained may not be applicable to other areas in Japan. Another limitation is that the questionnaire was self-administered; therefore, its accuracy is questionable.

Nevertheless, the present results provide useful information on CAM for patients with type 2 diabetes mellitus. The effects of CAM on health outcomes remain controversial [29]. Therefore, we may need to provide appropriate information on CAM to patients with type 2 diabetes mellitus. In addition, a longitudinal study is needed in the future to confirm the relationship between CAM and HRQOL in patients with type 2 diabetes mellitus.

## 5. Conclusions

Some patients with type 2 diabetes mellitus used CAM, and HRQOL was significantly lower in patients who used some CAM than in those without any CAM. Because of the limited evidence on the contribution of CAM to health outcomes, it seemed important to provide appropriate information about CAM to patients with type 2 diabetes mellitus.

## Figures and Tables

**Table 1:** Clinical profiles of patients with type 2 diabetes mellitus enrolled in this study.

	Total		
	Mean ± SD	Minimum	Maximum
Number of subjects	421		
Age (years)	67.3 ± 12.8	17.0	92.0
Height (cm)	161.8 ± 9.6	134.2	190.0
Body weight (kg)	66.7 ± 15.3	38.4	138.0
Body mass index (kg/m^2^)	25.3 ± 4.6	16.1	46.4
Duration of diabetes (day)	5863.9 ± 3711.7	0.0	17,854.0
Albumin (g/mL)	4.1 ± 0.5	2.0	5.3
Triglycerides (mg/dL)	190.4 ± 144.3	34.0	1630.0
LDL cholesterol (mg/dL)	103.9 ± 30.9	25.0	195.0
HDL cholesterol (mg/dL)	54.4 ± 15	9.0	105.0
HbA1c (%)	7.5 ± 1.5	4.0	13.5
Blood glucose (mg/dL)	165.9 ± 62.3	54.0	428.0
Serum creatinine (mg/dL)	1.0 ± 0.7	0.5	10.5
eGFR (mL/min/1.73m^2^)	61.3 ± 19.6	4.4	115.4
HRQOL	0.860 ± 0.200	−0.290	1.000
Use of Insulin (the number of subjects)	195 (46.3%)		
Use of oral hypoglycemic agents (the number of subjects)	342 (81.2%)		
Oral hypoglycemic agents: 1 Tablet	126		
Oral hypoglycemic agents: 2 Tablets	119		
Oral hypoglycemic agents: 3 Tablets	70		
Oral hypoglycemic agents: ≥4 Tablets	27		

SD: standard deviation. eGFR: estimated glomerular filtration rate. HRQOL: health-related quality of life.

**Table 2:** Use of complementary and alternative medicine in patients with type 2 diabetes mellitus.

	I Have Used and Still Use.		I Have Used, but I Do Not Use It Now.		I Have Never Used It.		I Do Not Remember and/or Do Not Know.	
	Subjects	%	Subjects	%	Subjects	%	Subjects	%
Supplements and/or Health Foods	112	26.6	56	13.3	248	58.9	5	1.2
Massage	49	11.6	68	16.2	299	71.0	5	1.2
Osteopathy	36	8.6	67	15.9	315	74.8	3	0.7
Spa therapy	19	4.5	11	2.6	386	91.7	5	1.2
Aromatherapy	6	1.4	8	1.9	402	95.5	5	1.2
Kampo	33	7.8	34	8.1	347	82.4	7	1.7
Acupuncture and/or Moxibustion	12	2.9	38	9.0	366	86.9	5	1.2
Yoga	10	2.4	14	3.3	393	93.3	4	1.0
Bone grafting	13	3.1	40	9.5	366	86.9	2	0.5
Chiropractic therapy	4	1.0	21	5.0	388	92.2	8	1.9
Magnetic therapy	9	2.1	10	2.4	396	94.1	6	1.4
Forest therapy	1	0.2	5	1.2	408	96.9	7	1.7
Music therapy	2	0.5	4	1.0	409	97.1	6	1.4
Thermotherapy	3	0.7	5	1.2	406	96.4	7	1.7
Qigong	2	0.5	2	0.5	410	97.4	7	1.7
Fasting therapy	3	0.7	6	1.4	407	96.7	5	1.2
Ayurveda	1	0.2	1	0.2	408	96.9	11	2.6
Homeopathy	2	0.5	1	0.2	408	96.9	10	2.4

**Table 3:** Comparison of health-related quality of life in type 2 diabetes mellitus patients with and without the use of CAM.

	CAM (+)		CAM (−)		*p*
	Subjects	Mean ± SD	Subjects	Mean ± SD	
Supplements and/or Health Foods	112	0.838 ± 0.217	309	0.869 ± 0.198	0.141
Massage	49	0.811 ± 0.235	372	0.868 ± 0.198	0.080
Osteopathy	36	0.825 ± 0.230	385	0.864 ± 0.201	0.273
Spa therapy	19	0.779 ± 0.221	402	0.865 ± 0.202	**0.029**
Aromatherapy	6	0.846 ± 0.122	415	0.861 ± 0.204	0.345
Kampo	33	0.780 ± 0.212	388	0.868 ± 0.201	**0.001**
Acupuncture and/or Moxibustion	12	0.849 ± 0.158	409	0.861 ± 0.205	0.470
Yoga	10	0.849 ± 0.175	411	0.861 ± 0.204	0.580
Bone grafting	13	0.756 ± 0.267	408	0.864 ± 0.200	0.094
Chiropractic therapy	4	0.673 ± 0.108	417	0.863 ± 0.203	**0.008**
Magnetic therapy	9	0.679 ± 0.255	412	0.865 ± 0.201	**0.007**
Forest therapy	1	0.796 ± −	420	0.861 ± 0.204	–
Music therapy	2	0.692 ± 0.105	419	0.862 ± 0.203	0.083
Thermotherapy	3	0.559 ± 0.273	418	0.863 ± 0.202	**0.016**
Qigong	2	0.898 ± 0.144	419	0.861 ± 0.204	0.998
Fasting therapy	3	0.617 ± 0.497	418	0.863 ± 0.200	0.280
Ayurveda	1	0.796 ± −	420	0.861 ± 0.204	–
Homeopathy	2	0.708 ± 0.124	419	0.862 ± 0.203	0.093

Comparisons were performed using the Mann–Whitney U test. CAM: complementary and alternative medicine. SD: standard deviation. Bold represents *p* < 0.05.

**Table 4:** Comparison of clinical profiles between type 2 diabetes mellitus patients with and without the use of CAM.

	CAM (+)	CAM (−)	*p*
Total	161	260	
Men	81	166	**0.006** ^c^
Women	80	94	
Age (years) *	66.6 ± 13.1 *	67.7 ± 12.5 *	0.398 ^b^
Body mass index (kg/m^2^) *	25.5 ± 4.8 *	25.3 ± 4.5 *	0.709 ^b^
Duration of diabetes (day) *	5807.0 ± 3412.6 *	5899.2 ± 3352.3 *	0.846 ^b^
Albumin (g/mL) *	4.1 ± 0.5 *	4.1 ± 0.4 *	0.488 ^b^
HbA1c (%) *	7.5 ± 1.6 *	7.5 ± 1.4 *	0.421 ^b^
eGFR (mL/min/1.73m^2^) *	59.8 ± 20.4 *	62.3 ± 19.0 *	0.212 ^a^

Comparisons were performed using unpaired *t* test (^a^), the Mann–Whitney U test (^b^) and χ^2^ test (^c^). CAM: complementary and alternative medicine. eGFR: estimated glomerular filtration rate. "*"represents mean ± standard deviation (SD). Bold represents *p* < 0.05.

## Data Availability

Not applicable.

## References

[1] Ministry of Health, Labor and Welfare in 2019, Japan https://www.mhlw.go.jp/stf/newpage_14156.html.

[2] The Japan Diabetes Society Fourth Five-Year Strategic Plan Against Diabetes Mellitus. http://www.fa.kyorin.co.jp/jds/uploads/plan_5years_4th.pdf.

[3] National Cancer Institute at the National Institutes of Health Complementary and Alternative Medicine. https://www.cancer.gov/about-cancer/treatment/cam..

[4] Zhang L., Cheng Y., Li H., Zhou Y., Sun B., Xu L. (2018). Meta-analysis of randomized controlled trials on the efficacy of daikenchuto on improving intestinal dysfunction after abdominal surgery. Ann. Surg. Treat. Res..

[5] Kono T., Shimada M., Nishi M., Morine Y., Yoshikawa K., Katsuno H., Maeda K., Koeda K., Morita S., Watanabe M. (2019). Daikenchuto accelerates the recovery from prolonged postoperative ileus after open abdominal surgery: A subgroup analysis of three randomized controlled trials. Surg. Today.

[6] Ishizuka M., Shibuya N., Nagata H., Takagi K., Iwasaki Y., Hachiya H., Aoki T., Kubota K. (2017). Perioperative administration of traditional Japanese medicine daikenchuto relieves postoperative ileus in patients undergoing surgery for gastrointestinal cancer: A systemic review and meta-analysis. Anticancer Res..

[7] Matsunaga S., Kishi T., Iwata N. (2016). Yokukansan in the treatment of behavioral and psychological symptoms of dementia: An updated meta-analysis of randomized controlled trials. J. Alzheimer’s Dis..

[8] Imai H., Takeshima N., Oda H., Chen P., Sawada E., Furukawa T.A. (2017). Choto-san versus placebo for patients with dementia: Systematic review and meta-analysis. Psychogeriatrics.

[9] Matsuda Y., Kishi T., Shibayama H., Iwata N. (2013). Yokukansan in the treatment of behavioral and psychological symptoms of dementia: A systematic review and meta-analysis of randomized controlled trials. Hum. Psychopharmacol..

[10] Shimoichi Y., Haruki J., Wakayama I. (2014). Acupuncture for patients with lower back pain -Systematic review of randomized trials conducted in Japan. J. Jpn. Soc. Acupunct. Moxibustion.

[11] Eisenberg D.M., Davis R.B., Ettner S.L., Appel S., Wilkey S., Van Rompay M., Kessler R.C. (1998). Trends in alternative medicine use in the United States, 1990–1997: Results of a follow-up national survey. JAMA.

[12] Clarke T.C., Black L.I., Stussman B.J., Barnes P.M., Nahin R.L. (2015). Trends in the use of complementary health approaches among adults: United States, 2002–2012. Natl. Health Stat. Rep..

[13] Misawa J. (2011). Influence of psychosocial factors on the use of complementary and alternative medicine. J. Health Welf. Stat..

[14] Ishibashi Y., Horiguti I., Kawaminami K., Kigawa M., Marui E. (2016). Use of Integrative Medicine in Japan. J. Health Welf. Stat..

[15] Yamashita H., Tsukayama H., Sugishita C. (2002). Popularity of complementary and alternative medicine in Japan: A telephone survey. Complement. Ther. Med..

[16] Fukuda S., Watanabe E., Ono N., Tsubouchi M., Shirakawa T. (2006). Use of complementary and alternative medicine and health problems. Jpn. J. Public Health.

[17] Mukai J., Kanamori A., Hiratsuka Y., Shimada K., Otori K., Koichiro Atsuda K. (2017). Overview of Complementary and Alternative Medicine in Diabetes Patients and Clinical Factors Affecting the Adoption of Food for Specified Health Uses: A Questionnaire-Based Survey. J. Jpn. Diab. Soc..

[18] Ministry of Health, Labor and Welfare in 2019, Japan Survey and Research on the Need for the Dissemination of Information on Integrative Medicine. https://www.mhlw.go.jp/stf/shingi/2r9852000002vsub-att/2r9852000002vsy2.pdf.

[19] Japanese EuroQol Translation Team (1998). The development of the Japanese EuroQol Instrument. J. Health Soc..

[20] Yapp L.Z., Scott C.E.H., Howie C.R., MacDonald D.J., Simpson A.H.R.W., Clement N.D. (2022). Meaningful values of the EQ-5D-3L in patients undergoing primary knee arthroplasty. Bone Jt. Res..

[21] Zare F., Ameri H., Madadizadeh F., Aghaei M.R. (2021). Validity and reliability of the EQ-5D-3L (a generic preference-based instrument used for calculating quality-adjusted life-years) for patients with type 2 diabetes in Iran. Diabetes Metab. Syndr..

[22] Yokoyama H., Hishida A. (2009). Collaborators developing the Japanese equation for estimated GFR. Revised equations for estimated GFR from serum creatinine in Japan. Am. J. Kidney Dis..

[23] Radwan H., Hasan H., Hamadeh R., Hashim M., AbdulWahid Z., Hassanzadeh Gerashi M., Al Hilali M., Naja F. (2020). Complementary and alternative medicine use among patients with type 2 diabetes living in the United Arab Emirates. BMC Complement. Ther. Med..

[24] Yıldırım D.İ., Marakoğlu K. (2018). Complementary and alternative medicine use amongst Turkish type 2 diabetic patients: A cross-sectional study. Complement. Ther. Med..

[25] Huo N., Ray G.E., Mehta S., LoBello S.G. (2015). Complementary and alternative medicine use among people with asthma and health-related quality of life. J. Asthma.

[26] Opheim R., Lie Høivik M., Bernklev T., Jelsness-Jørgensen L.P., Moum B. (2016). The Use of Complementary and Alternative Medicine among Patients with Inflammatory Bowel Disease Is Associated with Reduced Health-Related Quality of Life. Gastroenterol. Res. Pract..

[27] Rhee T.G., Pawloski P.A., Parsons H.M. (2019). Health-related quality of life among US adults with cancer: Potential roles of complementary and alternative medicine for health promotion and well-being. Psychooncology.

[28] Beatty L.J., Adams J., Sibbritt D., Wade T.D. (2012). Evaluating the impact of cancer on complementary and alternative medicine use, distress and health related QoL among Australian women: A prospective longitudinal investigation. Complement. Med. Ther..

[29] Lu C.L., Liu X.H., Stub T., Kristoffersen A.E., Liang S.B., Wang X., Bai X., Norheim A.J., Musial F., Alræk T. (2018). Complementary and alternative medicine for patients with chronic fatigue syndrome: A systematic review. BMC Complement. Altern. Med..

